# Supplementary material for: Exploring the Potential of a School-Based Online Health and Wellbeing Screening Tool: Young People’s Perspectives
Source: Int J Environ Res Public Health. 2022 Mar 29;19(7):4062. doi: 10.3390/ijerph19074062 (PMC8998184; doi:10.3390/ijerph19074062)
Supplement: Supplementary file 1 [file ijerph-19-04062-s001.zip › ijerph-1605742-supplementary.pdf]

File 1 Topic Guide

Topic guide: **Children and young people**

Note: This topic guide is indicative and will be subject to incremental change from emergent findings/information during the research. This is a guide to the topics to be covered during the interviews, it is not a script, and therefore the order of topics will be flexible.

Introduction:

- Thank you for participating
- Introduction of self
- introduction of study
- Key points
    - length of interview (30 minutes)
    - interview as a discussion which will cover key topics
    - no right or wrong answers - exploring perspectives
    - participation is voluntary - right to withdraw participation
    - confidentiality/anonymity
    - how findings will be reported
    - interview will be recorded
    - Inform that interviewer does not know status of young people in the programme
    - Questions? Happy to proceed? [Complete and sign consent form if face-to-face interview]

RECORD [obtain verbal consent if telephone/video interview]

<u>Digital Health Contract (DHC)</u>

What do you know about the DHC programme

- Can you tell me what you know about the DHC programme
    - what is does
    - how it works
- How has the programme been explained to you
    - by whom

- What is the online survey like to do
    - it is easy to follow and complete
    - How long did it take you to complete
    - What's it like filling out the DHC in school?
        - does it feel private - where/when did you do it
        - do you think doing it this way changes people answers - does it yours
        - do you think there are better ways to do it

What do you think about the questions [**have examples**]

- does the language / terminology make sense
- are the areas the questions cover relevant
- do you think there is anything missed or anything that could be added

What do you think about the health messages in the DHC? [**have examples**]

- are they relevant - do they give you information you need/find helpful
- do they make sense/easy to understand

        - could they be improved
        - do you think people are likely to listen to them/use them
            - why is this

Do you think the DHC is a good way to talk about health issues?

- do you think people are honest when completing DHC (to talk about health issues)
    - Why is this?
    - what about you?

    - Do you think that being honest when answering some questions is harder for some people
        - which questions
        - for whom

- do you think people find it better to talk to a teacher/nurse/professional face-to-face or doing a online survey
        - which do you think they would be more honest with
- What do you think can stop people talking about any health issues they are having
        - contacting staff / arranging appointments
    - what kind of things can make people more likely to talk about health issues

    - What would be the best way to get people to talk about any problems they might be having? (online / face-to-face / phone)

What do you think/know about what happens to the responses in the survey?

- What do you think the school does with the survey information?
- some young people are 'red flagged', what does this mean to you?
    - what do you think a red flag is
    - what do you think about this
    - How do you think people may feel if they were red flagged

- how do you feel about school nurses potentially contacting you after to discuss your responses?
    - what do you think this would mean
    - [don't provide personal details] do you know anyone this has happened to

    - do you think people would find it easier for a nurse to contact them, rather than them to contact a nurse
    - What do you understand about the role of the school nurse
        - Why do you think young people see the school nurse?

How do you think people would feel about seeing a school nurse for a support session following the DHC?

    - [don't provide personal details] do you know anyone this has happened to

<u>Reflections</u>

What is good about the DHC programme

What could be improved with the DHC

- what could be done differently

What made you want to take part in this interview today?

Thank and finish

Anything else you would like to add

File 2 Coding Framework

**Understanding of DHC**	Purpose
	Process
	How was it explained
	'Red flag' wording
**Experience completing DHC**	Usability
	Question complexity/understanding
	Question language/wording
	Question content/scope
	Perceptions of privacy
	Relevance of questions / of health messages
**Perceptions of DHC usefulness**	Perceived/reported validity in responses
	Online/face-to-face preference
	School nurse involvement
	General perceptions
**Support seeking**	Barriers/facilitators
	Options for support
**School nursing**	Perceptions of role
**Suggested improvements**	

File 3 Information Sheet

Information sheet for young people

Evaluation of the Digital Health Contact

We are from the University of Sheffield and we would like to ask you to take part in a research project about the Digital Health Contact (DHC) programme at your school. Before you decide if you want to join in, it's important to understand what it will involve. So please read this information sheet carefully and talk to your parents or carer about it if you want.

Key points

- You are being invited to take part in an interview at your school which will last around 30 minutes
- The interview is about what you think about the Digital Health Contact (DHC) you've completed at your school
- You will get a £10 shopping voucher for taking part in the interview as a thank you for your time
- What you say in the interview will be kept confidential, that means we will not tell anyone what you have said. The only time we may have to tell someone what you have said is if you say something which suggests there is a risk of significant harm to someone (including you)
- You do not have to take part if you do not want to, and can change your mind about taking part at any point
- If you want to take part just let your teacher/ the school nurse team know, and an interview time and place will be set up for you

Why are we doing this research?

The Digital Health Contact (DHC) is an online health and wellbeing questionnaire which you have recently completed at school. Everybody who does the questionnaire is given advice on how to stay healthy, and some people will be offered extra help from a school nurse or other healthcare specialists.

We're looking at the views of different people (students, parents, teachers) to see what people think about the DHC. Your views, as the people who actually do the DHC, are really important. We're not asking about the answers you gave in the DHC survey, but about what you think of the DHC more generally, and if you think it's a good way to talk about your health. You will be asked questions on what is good about it and what could be done to improve it. It does not matter if you feel you do not know much about the DHC, we are interested in your thoughts and perspectives. We will also be asking your parents and some of your teachers to take part in an interview about the DHC.

We hope that this will help us to understand ways to better deliver support and improve young people's health.

The study is led by the Universities of Sheffield and Bristol, and is funded by the National Institute for Health Research (NIHR) School for Public Health (https://sphr.nihr.ac.uk/).

Do I have to take part?

Taking part in the interview is entirely up to you. You do not have to take part if you do not want to.

If you do take part you will be invited to an interview with someone from our research team. This interview will take place in your school in a private room. The interview will be either face-to-face with a researcher, or over the phone or on an online video call, depending upon safety and which is most appropriate due to social distancing requirements. With your permission, the interview will be audio recorded as it is important that we remember what you say to us. If the interview is on an online video call, it will be audio recorded only, there is no video recording.

If you want to take part, let your teacher or school nurse team know and everything will be organised for you.

The interview will last about 30 minutes.

We will ask you to complete a consent form before participating to make sure you are happy to take part, and that you understand what taking part involves.

You will get a £10 shopping voucher for taking part in the interview as a thank you.

If you change your mind about taking part that is okay. You do not have to take part if you do not want to. If you change your mind, you can withdraw from the study at any time without giving a reason. This includes after the interview has taken place. To withdraw from the study you should contact one of the research team (contact details are at the end of this information sheet). If the interview has taken place, you can withdraw from the study up to one week after completion of the interview. After this date it will not be possible to withdraw your interview from the study, due to any personal information linking it to you being removed, but we will delete any personal details we have about you.
If you do want to withdraw from the study, any personal details we have about you will be deleted.

Are there any possible benefits or downsides to taking part?

We hope that you will enjoy discussing the DHC with us and enjoy having the chance to put forward your thoughts and opinions about how the DHC works, and, hopefully, making it work better.

We will not ask you about the answers you gave in the DHC survey. We will only ask you about your thoughts around the DHC programme more generally. We will ask for example, how you think people may feel if they were 'red flagged' in the DHC. We understand some of the questions may be around sensitive topics, so it is important to know that you do not have to answer any questions you do not want to. If you feel uncomfortable in any way or decide you no longer want to take part, then you can stop at any time without giving a reason. Also, you can skip questions and you do not have to answer a question if you do not want to.

Is taking part confidential?

What you say in the interview will be kept confidential, that means we will not tell anyone what you have said. The only time we may have to tell someone what you have said is if you say something which suggests there is a risk of significant harm to someone (including you). If this happens we will talk about what will happen next.

Any personal information we collect about you during the research will be kept strictly confidential and will only be accessible to members of the research team. We'll have to record some personal details about you so we can set up the interview (your name, your school), but this will be securely stored in a password protected spreadsheet on the University of Sheffield's systems, and these details will be deleted as soon as possible after your interview. We will write up what you tell us to help improve the DHC, but nothing will be linked back to you. You will not be named or otherwise identified at any point.

All interviews will be audio recorded using a digital recorder. Afterwards your interview will be typed up on a computer with all personal information removed so you can't be identified. All copies of the recording will be destroyed one month after being typed-up. Typed-up interviews will be kept securely in restricted folders on password protected computers that are only accessible by the research team. After the project we will also store the typed up interview securely online in a data repository called ORDA; a data repository is a place where research information can be kept after a project has finished for other people to use. This means that other researchers may read the typed up interviews in the future, but they will not have any way of linking this to you as all personal information will be removed.

Consent forms will be scanned onto and stored securely in a restricted folder on the University of Sheffield's system. Any paper copies of consent forms will then be destroyed by shredding and confidential waste disposal. Consent forms will be securely stored for 10 years and then deleted.

Who is responsible for looking after my information?

The University of Sheffield will act as the Data Controller for this study. This means the University of Sheffield is responsible for looking after your information and using it properly.

What is the legal basis for processing my personal data?

Data protection laws say we have to explain to you why we are asking for the personal information we will collect in the interviews and what we will do with it. We need this information for research that is a task in the public interest – this means something that is good for the general public.

What will happen to the findings of the research?

The results will be written up so that other people, researchers, government agencies, and the public can learn about the DHC and ways to improve young people's health.
Your name will not be used in any reports or presentations from this project.

Has anyone checked this project is okay?

Before any research is allowed to happen it has to be checked by a group of people called an Ethics Committee. They make sure the research is okay to do. This project has been checked by the University of Sheffield Research Ethics Committee through the School of Health and Related Research.

What if I have any problems?

If you have any questions or are unhappy about anything, please let Nick Woodrow or another member of the research team know (contact details are listed at the end of this information sheet). If you would prefer to talk to someone outside of the research team, you can contact Professor John Brazier, Dean of the School of Health and Related Research, (0)114 222 0726, j.e.brazier@sheffield.ac.uk

If you have any issues about the way we handle your personal data, you can contact the University's Data Protection Officer, Luke Thompson on 0114 2221117 or dataprotection@sheffield.ac.uk. If you are not satisfied with how your complaint is handled, you may then escalate the complaint to the ICO (Information Commissioner's Office).

How can I find out more about this project?

If you would like to ask any questions about this project, you can contact Nick Woodrow on 0114 2226399 or email him at n.woodrow@sheffield.ac.uk or Dr Hannah Fairbrother on 0114 222 2044 or email her at h.fairbrother@sheffield.ac.uk

Thank you very much for reading this!
